# The Visit of Wilhelm Herbst

**Published:** 1886-08

**Authors:** 


### THE VISIT OF WILHELM HERBST.

American dentists are a matter-of-fact body of men, and whatever appeals to the practical side of their natures meets with a warm reception. As a class they are not as well versed in theory as are their brethren of England or Germany. They have made comparatively little advancement in general science, and in dental technics they cannot compete with many of other nationalities. All men are the creatures of circumstances, and their development is dominated by their surroundings. America is a comparatively new country, and Americans so far have been obliged to wage a ceaseless warfare with the forces of nature. The face of our land must first be remoulded, and the country subdued and improved. All this has developed the practical side of American character, and made us the most ingenious and inventive people in the world. We have been, during the whole period of our national history, engaged in solving practical problems, and the consequence is a patent office full of the models of ingenious mechanisms, the records of inventive triumphs which have astonished the world.

American dentistry is a synonym for clever devices, for unrivalled manipulative triumphs, for surgical and operative skill that is unequalled. But we are not a scientific profession, nor do we excel in the patient assiduity which constructs a beautiful piece of mechanism after a definite mechanical rule. We are apt to scoff at the general attainments of foreign dentists who have an acknowledged standing in the scientific world, and to deride as only an artisan the trained mechanic who spends his life in perfecting details. There is a happy medium that we shall sometime reach, but in the meantime our colleges, which are chiefly engaged in teaching practical dentistry, suffer in the eyes of foreign dentists, who refuse to acknowledge them because they are more practical than literary and scientific.

It is this peculiar natural tendency, or characteristic of American dentistry, that has caused American dentists to warmly welcome Wilhelm Herbst to their midst. Here was a man who appealed very strongly to several phases of American character. He had devised or discovered a new way to reach a practical end sought by every American dentist. He was a mechanical genius, an inventor, and as such had attained to the summit of the highest ambition of every American. He appealed as strongly to American sympathies as he would have done to Germans had he devised a new philosophy, to Englishmen by opening up another industry or discovering an original law of trade, or to Frenchmen by inventing a new soup.

There was a kind of suspicion too, that he had not received exactly fair play at home. Either his methods or his personality were such as not to commend him to the steady-going Germans, and the idea has taken possession of the American mind that he had been whistled down the wind, without a careful examination of that which he had to present. The Germans are great sticklers for professional etiquette, and for the ethics of dentistry. We are careless of these things (altogether too careless), and are ready to accept what we believe to be a good thing, no matter from what irregular source it may come. It was believed here that Americans were better judges of his practical methods than were Germans, because we are a more practical people.

The chivalrous feelings of Americans were aroused, not only by these considerations, but by the fact that Herbst had sufficient confidence in his ideas to place their exemplification before American dentists, and to risk their verdict. His enthusiasm appealed to enthusiastic natures, for Americans love an earnest man, even though he may be wrong. The fact that he was willing to make the great sacrifice demanded by a trip to this country for the purpose of teaching Americans something in their own line, also strongly appealed to their gratitude. It was the first time that a foreigner had ever visited our shores with such a benevolent object in view, and American dentists desired to testify to the world that they were always anxious to learn, and grateful to those who could and would teach them anything. Many of our dentists had visited the old world with what they believed to be valuable ideas and methods, but because our ways were not their ways, and because the information was not offered through the conventional channels, they had received but a cold welcome. It is undoubtedly the case that many competent operators who have gone to Europe from here have violated some of the proprieties of their professional life, but it should be remembered that we as a people are not so conventional as are they, and our ways are those which are supposed to be the most direct. It astonishes Englishmen and dentists of other nationalities when they visit our society meetings and witness our free-and-easy, unceremonious methods. A little infusion of our directness and earnestness would, we think, be beneficial to foreign societies and meetings, and it is certain that an importation of some of their decorum and high regard for professional ethics would greatly redound to our good.

Well, we did not set out to write a disquisition on American or foreign professional character, but to consider why Herbst was so warmly received in this country. In this number will be found a full account of what transpired at the festivities in New York. It is certain that the rotary method of filling teeth will here receive a thorough and careful test. Indeed, it was not entirely new to Americans, for Dr. Bödecker, a dentist in whose ability and honesty every American dentist has the utmost confidence, had already introduced the method here, and this journal had devoted much space to its consideration. The method had not, however, made any considerable progress, but it was felt that it was only fair that a verdict should not be pronounced until its exemplification at the hands of its discoverer had been witnessed.

Herbst has now been here, and has made a very favorable impression by his geniality, his earnestness, and his evident great mechanical ability. His methods and ideas remain with us, and must make their way by their own merits, if they advance at all. But even though not an American dentist may adopt the method, the visit of Herbst will not have been in vain, for there has been a broadening of ideas, a professional development through this opportunity for comparing American manipulative skill with that of a capable exponent of another country. Whatever may be thought of the merits of the system of burnishing gold into cavities in teeth, there seems to be but one opinion concerning the innate operative ability of its inventor.

It should be noticed that the report of the Herbst festivities in New York does not trench upon the regular matter of this number. The manuscript was not received until the number had been made up, and it could only be admitted by enlarging the size of the journal. This has accordingly been done, and the report is, therefore, entirely extra matter, beyond the usual fifty-six pages.

### THE A. D. A. MEETING.

This number is issued a few days in advance of the usual time, that its announcements may be in the hands of subscribers before the time for the meeting at Niagara. It is expected that a large number will be in attendance at that meeting. Every arrangement possible has been made by the committee, and an unusually large number of papers of merit are already promised. The indications now are that even more matter will be presented to some of the sections at least, than there will be time and opportunity to report to the general meeting. Niagara is now free to the people of America, and under the new regime it is the most delightful place of resort that we have. The hall for the meeting is the new theatre at Niagara, and it offers unusual advantages. The headquarters for the Association will be at the International Hotel, which promises reduced rates to the members. The Cataract House, which has heretofore been the home of many delegates during our meetings, seems to entertain the idea that because its attractions are lessened its prices should be advanced. The improvements made, and the opening of the National Park, have robbed that old house of its river parlors and of its best rooms, but it refuses to make any concessions for delegates and members.

Those who desire accommodations quite as good, but at a less price than those of the International, can obtain them at the Prospect Park Hotel. Members will be received here at $2.00 per day, by engaging their rooms in advance, and they will be certain of good entertainment.

